# The Impact of Leveraging an Emergency Department Observation Unit for Pandemic Response on Observation Outcomes: A Retrospective Observational Difference-In-Differences Study

**DOI:** 10.1016/j.acepjo.2026.100471

**Published:** 2026-07-28

**Authors:** Michael J. Carr, Iyesatta M. Emeli, Douglas W. Chesson, George B. Hughes, Marta A.W. Rowh, Ingrid B. Bloom, Tim P. Moran, Michael A. Ross

**Affiliations:** Department of Emergency Medicine, Emory University School of Medicine, Atlanta, Georgia, USA

**Keywords:** emergency department observation unit, public health emergency, hospital length of stay, health care costs, disaster preparedness

## Abstract

**Objectives:**

It is unknown what the impact of leveraging an emergency department observation unit (EDOU) for alternative purposes during a public health emergency might be. The study objective was to determine the impact of shifting observation patients from an EDOU to inpatient beds on hospital observation outcomes—length of stay (LOS), total cost, and inpatient admission rate.

**Methods:**

This is a retrospective observational difference-in-differences study across 4 hospitals within a large academic health system in Atlanta, Georgia. All had type 1 EDOUs managed by the department of emergency medicine. At the onset of the pandemic, the intervention hospital converted the EDOU to a COVID unit, displacing all observation patients to inpatient beds (April 2020). The remaining 3 hospitals maintained operations and served as controls. Data were abstracted from hospital clinical and financial observation databases. Comparisons were made between the preintervention period (August 2019 to March 2020) and the intervention period (April 2020), and between intervention and control hospitals. The primary outcomes included: average per patient cost, total (observation + inpatient) LOS, observation LOS, and inpatient admission rate. Outcomes were evaluated using mixed-effects gamma regressions and logistic regressions. Analyses were adjusted for age, sex, triage level, diagnosis, and procedural International Classification of Diseases codes. We present mean ratios (MRs) for continuous outcomes, odds ratios for binary outcomes, and 95% CIs. The difference-in-differences effect was tested using a time-by-site interaction term.

**Results:**

Over the study period there were 22,263 observation patients (3080 intervention patients, 19,183 control patients). Average age was 58 (43 to 72), and 56% were female. Five most common Clinical Classification System (CCS) conditions were chest pain (10.8%), hypertension (5.4%), fluid electrolyte disorders (3.7%), syncope (2.5%), and abdominal pain (2.4%). The difference-in-differences interaction was significant for all outcomes. Total cost for the control hospitals remained stable ($3876.6 vs $3997.1; MR =1.03, 95% CI: 0.99-1.08) but increased from $3449.1 to $4645.5 (MR = 1.35, 95% CI: 1.20-1.52) for the intervention hospital. For total LOS, control hospitals saw a significant decrease in LOS (36.4 h vs 34.3 h, MR = 0.94, 95% CI: 0.90-0.99); the intervention hospital saw a nonsignificant increase (32.1 h vs 36.6 h, MR = 1.14, 95% CI: 1.00-1.30). For only observation LOS, control hospitals saw a significant decrease (23.8 h vs 19.8 h; MR = 0.83, 95% CI: 0.79-0.87), whereas the intervention hospital saw a significant increase (21.3 h vs 26.1 h; MR = 1.22, 95% CI: 1.09-1.38). Finally, for admission rates, control hospitals remained stable (16.4% vs 16.8%; odds ratio of 1.02, 95% CI: 0.88-1.19), whereas the intervention hospital saw an increase from 13.3% to 21.0% (odds ratio of 1.74, 95% CI: 1.16-2.61). The findings were similar when studying only discharged patients.

**Conclusion:**

Displacing EDOU observation patients to inpatient beds to accommodate disaster or surge patients is associated with increases in observation patient cost, LOS, inpatient bed use, and inpatient admission rate.

**The Bottom Line**

Emergency department observation units are often repurposed during disasters, but the impact on patient outcomes is not well understood. In this retrospective study of 22,263 patients across 4 hospitals, one hospital converted its observation unit into a COVID-19 unit and shifted patients to inpatient beds, while 3 hospitals did not. At the intervention hospital, average cost increased from $3,449 to $4,646, observation length of stay increased from 21.3 to 26.1 hours, and admission rates rose from 13.3% to 21.0%, while control hospitals remained stable or improved. These findings suggest that repurposing observation units may worsen efficiency, increase costs, and strain inpatient resources during public health emergencies.

## 1 Introduction

### 1.1 Background

Emergency department observation units (EDOUs) typically provide short-term acute care and monitoring, ideally for less than 24 hours post-ED visit, to patients requiring extended observation but not hospital admission. Type 1 EDOUs are defined by their use of protocol-driven care within dedicated spaces.^1^ During disasters or pandemics, these units can be repurposed to manage patient surges, isolate contagious individuals, or address resource constraints. However, the clinical and operational impacts of repurposing EDOUs during a public health crisis remain poorly understood.

### 1.2 Importance

The COVID-19 pandemic exemplifies the biological disaster type, which led to unprecedented demands on health care infrastructure, such as decreased inpatient bed availability, ED crowding, and unparalleled infrastructural demands.^2^, ^3^, ^4^, ^5^ Prior research has demonstrated the flexible utility of EDOUs in managing disaster scenarios, but empirical data quantifying their impact on observation outcomes during a pandemic are lacking.^6^, ^7^, ^8^

### 1.3 Goals of This Investigation

Our objective was to evaluate the impact of shifting observation patients from an EDOU to inpatient beds during the COVID-19 pandemic on key hospital observation outcomes, including patient length of stay (LOS), total cost, and inpatient admission rate.

## 2 Methods

This was a retrospective observational difference-in-differences (DID) study conducted within a large academic health system in Atlanta, Georgia.^9^ The study included one intervention hospital, Emory Johns Creek Hospital (EJCH), a community hospital that repurposed its type 1 EDOU into a COVID-19 inpatient unit in April 2020. The hospital has a 20-bed hybrid observation unit that was built to accommodate EDOU patients (10 beds) and elective scheduled procedure patients (10 beds). It was located adjacent to the emergency department and separated from the rest of the hospital, making it an ideal setting for an isolated COVID-19 unit. Shortly after the onset of the COVID-19 pandemic all observation services and scheduled procedure services were moved into the hospital to a type 4 observation setting, creating a 20-bed COVID patient unit capable of managing medical/surgical floor patients and intensive care unit patients. Control hospitals were 2 teaching hospitals (Emory University Hospital [EUH], and Emory University Hospital Midtown [EUHM]) and one community hospital (Emory Saint Joseph’s Hospital [ESJH]), all maintaining standard type 1 EDOU operations. All EDOUs used the same EDOU operational guideline, protocols, order sets, and EDOU staffing models. Hospitals varied in terms of the percentage of hospital observation patients captured in the EDOU and the number of EDOU beds (Table S1).

We included patients managed under observation status from August 2019 through April 2020. The COVID patient subgroup was not within the scope of this analysis because they were not observation patients. This study included observation of patients managed in the EDOU (Type 1 setting), as well as observation of psychiatric patients who were at risk of self-harm and managed by observation protocol but physically located in the ED for patient safety reasons (Type 3 setting).

At EJCH, a 20-bed hybrid observation unit had been built to accommodate EDOU patients (10 beds) and elective scheduled procedure patients (10 beds). This study did not include scheduled elective outpatient procedure patients, because this group is different from emergency or observation patients. The emergency department did not manage the displaced observation patients who were moved to an inpatient bed. Patients were managed by respective inpatient services, such as hospital medicine. High efficiency particulate air filtration units were installed in each room. Existing doors to the unit and rooms provided further isolation. Several months later, as the pandemic progressed, the care of COVID inpatients was gradually returned to the hospital inpatient locations, and ED observation patients returned to the hybrid EDOU location.

This study was deemed exempt by the Emory University Institutional Review Board as it involves retrospective analysis of deidentified patient data, collected as part of routine clinical care, posing minimal risk to patient privacy and safety. This study adhered to the Strengthening the Reporting of Observational Studies in Epidemiology observational study design criteria.^10^

The primary outcomes measured were the percentage of observation patients captured in EDOUs (%EDOU), total patient cost, total LOS, observation-specific LOS, and inpatient admission rates. Total patient cost encompassed all associated facility clinical, and operational expenses incurred during the observation encounter and did not include professional costs. Total LOS included the cumulative duration from ED presentation to final discharge from observation or inpatient admission, which included inpatient length of stay for those patients who were admitted. Observation-specific LOS (Obs-LOS) was calculated as the time specifically spent in observation status, for both admitted and discharged patients. Inpatient admission rate was determined as the proportion of observation patients subsequently admitted to inpatient services. Confounding variables, such as age, sex, primary CCS diagnosis, triage acuity level, and procedural International Classification of Diseases codes, were also abstracted and controlled for in the analysis to adjust for potential variability in patient severity and complexity. Clinical and financial data were extracted from Cerner Clinical Database and EPSi financial cost accounting software (Enterprise Performance Systems Incorporated).

DID analyses were employed to control for biases and isolate pandemic effects. A subgroup analysis of only community hospitals was performed to compare similar hospital subgroups.

Sample size was driven by the period where the unit was entirely converted to a COVID-19 isolation unit (psychiatric observation patients remained outside of the EDOU, in the ED). Thus, the sample size was fixed. It is possible to estimate the magnitude of the interaction term that could be detected with sufficient (80%) power, given the sample size. These coefficients correspond to the degree to which the pre-to-post change in the intervention hospital is larger/smaller than the pre-to-post change in the control hospitals (eg, a value of 1.25 would indicate that the present study would have sufficient power to determine that the pre-to-post change in the intervention hospital was 25% larger than the pre-to-post change in the control hospitals). This calculation determined that the present study had sufficient power to detect interaction terms of 1.31, 1.21, 1.47, and 1.70 for cost, total LOS, Obs-LOS, and admission rate, respectively.

Continuous variables were described using medians and interquartile ranges (IQR) and categoric variables were described using frequencies and percentages. The outcomes of interest for the present study were total cost, total LOS in hours, observation of LOS (Obs-LOS) in hours, and the rate of hospital admissions. The continuous outcomes are positively skewed, bound by 0, and heteroscedastic. To evaluate these outcomes, we used a mixed-effects gamma regression with a log link function. We present mean ratios (MRs), 95% CIs, and *P*-values. For admission rate, we used a mixed-effects logistic regression. We present odds ratios (ORs), 95% CIs, and *P*-values. The mixed-effects models were used to account for clustering within the site. The DID comparison evaluates whether the change over time differs across control and intervention hospitals (ie whether the slope differs across sites). This is accomplished by including a site (intervention vs control) by time (pre vs post) interaction term in the regressions described above. A significant interaction term indicates the degree of change over time differed across sites. For significant interactions, we present the MR and/or OR separately for control and intervention sites. The regressions were adjusted for the following a priori covariates: age, sex, primary CCS diagnosis, triage acuity level, and procedural International Classification of Diseases codes. Natural cubic splines were used to allow for non-linear effects of age. Initial testing found no significant deviations from the parallel trends’ assumption.^11^ Analyses were conducted using R (v 4.3; R Core Team).

A DID study, such as this one, is an extension of preintervention/postintervention studies which also include control sites which did not receive the intervention/exposure. The change over time observed in the intervention site is then compared to the change over time observed in the control site(s). To further reduce bias, we also controlled for a priori covariates (described in the analysis section) and accounted for within-site clustering. The basic DID regression equation took the form of:

y=intercept+β1∗D+β2∗Site+β3∗D∗Site+Δ∗C+ϵ

where y is a given outcome, D is an indicator variable denoting whether a patient’s care took place before or after the conversion of the observation unit to a COVID-19 unit, Site is an indicator denoting whether the patient arrived at a control hospital or intervention hospital, the β values refer to the coefficients of interest, C is the set of covariates (eg, age, triage acuity etc.), Δ refers to the coefficients for the covariates, and *ϵ* refers to the model error. A DID effect is primarily inferred from a significant β3 term—that is, a significant interaction between time-period and site.

## 3 Results

A total of 22,263 observation patients (3,080 intervention hospital and 19,183 control hospitals) were included. Most patients (55.8%) were female, and the median age was 58 (IQR: 43-72). A total of 5 289 (23.8%) were admitted to the hospital, and 16,974 (76.2%) were discharged. The median total cost, total LOS, and Obs-LOS were $2574 (IQR: $1709 – $4400), 22 hours (IQR: 14 – 44), and 20 hours (IQR: 12 – 29), respectively. The most common (10.8%) CCS was nonspecific chest pain. Patient characteristics are presented in Table 1. Across all hospitals for the entire study period, 6.4% of patients had a psychiatric diagnosis and were managed in the ED (Type 3 setting).

**Table 1:** Patient characteristics of the entire study population.

Characteristic	N or M	% or IQR
Age, M / IQR	58	43 – 72
Sex, N / %		
Female	12,425	55.8
Male	9838	44.2
Admitted, N / %	5289	23.8
LOS, M / IQR	22	14 – 44
Obs-LOS, M / IQR	20	12 – 29
Total Cost, M / IQR	2574	1709 – 4400
10 Most Common CCS^a^, N / %		
Nonspecific chest pain	2409	10.8
Hypertension w/ complications	1206	5.4
Fluid and electrolyte disorders	815	3.7
Syncope	560	2.5
Abdominal pain and other digestive/abdomen signs and symptoms	525	2.4
Diabetes mellitus with complication	493	2.2
Urinary tract infections	436	2.0
Cardiac dysrhythmias	426	1.9
Coronavirus	420	1.9
Coronary atherosclerosis and other heart disease	391	1.8

CCS, Clinical Classification System, LOS, length of stay.

a 10 most common conditions represent 34.5% of total population.

### 3.1 Full Sample

**Figure 1:**
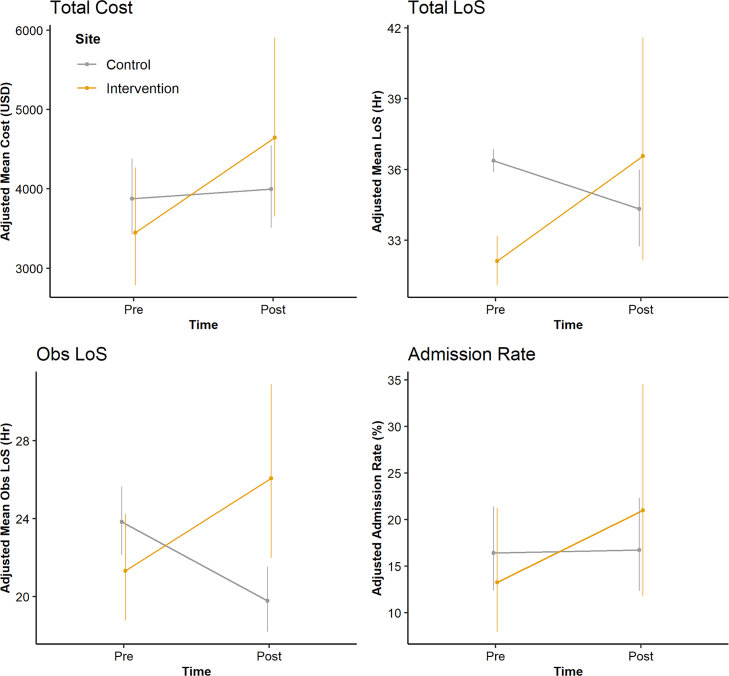
Hospital outcomes for all patients.

Adjusted outcomes are presented in Table 2 and accompanying Figure 1. The interaction term for total cost was significant (*P* <.001). This indicated that cost for the control hospitals remained stable ($3876.6 vs $3997.1; MR =1.03, 95% CI: 0.99-1.08) but increased from $3449.1 to $4645.5 (MR = 1.35, 95% CI: 1.20-1.52) for the intervention hospital. For total LOS, the interaction term was also significant (*P* =.008). Control hospitals saw a significant decrease in LOS (36.4 h vs 34.3 h MR = 0.94, 95% CI: 0.90-0.99); the intervention hospital saw a nonsignificant increase (32.1 h vs 36.6 h, MR = 1.14, 95% CI: 1.00-1.30). The interaction term was significant for Obs-LOS (*P* <.001). This indicated that control hospitals saw a significant decrease in Obs-LOS (23.8 h vs 19.8 h; MR = 0.83, 95% CI: 0.79 to 0.87), whereas the intervention hospital saw a significant increase (21.3 h vs 26.1 h; MR = 1.22, 95% CI: 1.09-1.38). Finally, the interaction term was significant for admission rates (*P* =.02). For the control hospitals, admission rates remained stable (16.4% vs 16.8%; OR = 1.02, 95% CI: 0.88-1.19). For the intervention hospital, however, admission rates increased from 13.3% to 21.0% (OR = 1.74, 95% CI: 1.16-2.61).

**Table 2:** Adjusted study outcomes.

Outcome	Control		Intervention		Difference-in-differences (95% CI)	*P* interaction
	Before	After	Before	After		
All patients						
Total Cost ($USD)	3876.6 (3428.9; 4382.7)	3997.1 (3512.2; 4548.9)	3449.1 (2786.9; 4268.8)	4645.5 (3651.1; 5910.7)	1075.8 (568.2; 1583.4)	<.001
Total LOS (hours)	36.4 (35.9; 36.9)	34.3 (32.7; 36.0)	32.1 (31.1; 33.2)	36.6 (32.1; 41.6)	6.5 (1.6; 11.4)	.008
Observation LOS (hours)	23.8 (22.1; 25.7)	19.8 (18.2; 21.5)	21.3 (18.8; 24.2)	26.1 (22.0; 30.9)	8.8 (5.9; 11.7)	<.001
Admission (%)	16.4 (12.4; 21.4)	16.8 (12.3; 22.3)	13.3 (8.0; 21.3)	21.0 (11.8; 34.5)	7.4 (1.3; 13.5)	.02
Discharged patients						
Total Cost ($USD)	2573.6 (2296.2; 2884.5)	2493.4 (2210.3; 2812.7)	2362.2 (1940.6; 2875.4)	3342.8 (2668.6; 4187.2)	1060.7 (718.0; 1403.3)	<.001
Total LOS (hours)	23.3 (21.9; 24.8)	19.3 (17.9; 20.8)	21.2 (19.1; 23.7)	24.8 (21.1; 29.2)	7.6 (4.6; 10.6)	<.001
Observation LOS (hours)	23.5 (22.2; 24.9)	19.5 (18.1; 21.0)	21.4 (19.3; 23.7)	25.2 (21.5; 29.6)	7.8 (4.7; 10.9)	<.001

LOS, length of stay.

### 3.2 Only Discharged Patients

**Figure 2:**
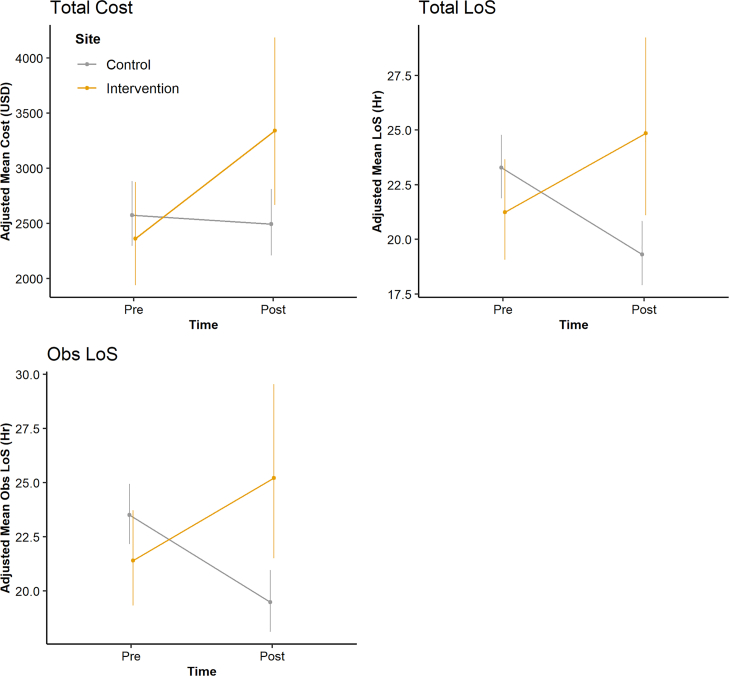
Hospital outcomes for discharged patients.

We conducted the same analyses (with the exception of admission rates) using only patients discharged from the observation status across all settings (Figure 2). In this subgroup, all 3 interaction terms were significant (*P* <.001). As with the full sample, total costs remained stable for control hospitals ($2573.6 vs $2493.4; MR = 0.97, 95% CI: 0.93-1.01) and significantly increased for the intervention hospital ($2362.2 vs $3342.8; MR = 1.42, 95% CI: 1.26-1.59). Total LOS significantly decreased for control hospitals (23.3 h vs 19.3 h; MR = 0.83, 95% CI: 0.79-0.87) and significantly increased for the intervention hospital (21.2 h vs 24.8 h; MR = 1.17, 95% CI: 1.03-1.33). Finally, Obs- LOS significantly decreased for control hospitals (23.5 h vs 19.5 h; MR = 0.83, 95% CI: 0.79-0.87) and significantly increased for the intervention hospital (21.4 h vs 25.2 h; MR = 1.18, 95% CI: 1.04-1.34).

Community hospital subgroup analysis: The primary analyses was repeated for only the 2 community hospitals, EJCH and ESJH, in the sample. The results are presented in Table S2. Cost significantly increased for both hospitals from pre-to-post (*P* <.001). However, the interaction was significant (*P* =.03) indicating that the increase in cost (indicated by the mean ratio) was significantly larger for EJCH than for ESJH. For total LOS, there was a significant increase from pre-to-post for EJCH (*P* =.01) but not for ESJH (*P* =.23). The interaction was nonsignificant (*P* =.08), indicating that the increases observed were similar across hospitals. Finally, there was a significant increase in discharged observation patient LOS for EJCH (*P* <.001) but a significant decrease for ESJH (*P* =.01). The interaction was significant (*P* <.001), indicating that the prechange to postchange (ie, MRs differed across hospitals).

## 4 Limitations

Limitations include potential residual confounding despite rigorous adjustments, single-system analysis impacting generalizability, and observational design precluding causal inference. Nevertheless, the robustness of the DID approach strengthens confidence in the findings. As a single-system study, the findings may not be fully generalizable to other settings, where variability in payer structures, available resources, and operational workflows may influence both the feasibility of observation unit displacement and its impact on patient outcomes.

As with any observational study of observation medicine, selection bias favoring patients managed in the EDOU is a potential limitation. However, this issue has been examined previously within the same health system using a propensity-weighted analysis designed to address selection bias. In that study, outcomes including LOS, cost, admission rate, and adverse events remained significantly different after adjustment, favoring EDOU care over nonobservation unit (NOU) settings.^12^ The current study was not designed to replicate those findings but rather to provide new insights into how EDOUs may be leveraged during disaster or surge conditions.

## 5 Discussion

Fully repurposing a type 1 EDOU for a pandemic or disaster was associated with compromised hospital observation operations, inpatient resource utilization, costs, and admission rates. There is a paucity of literature addressing the role of the EDOU in disasters. This study examines one specific function: the displacement of observation patients from the unit and the subsequent impact on patient outcomes. Our findings demonstrate that repurposing an EDOU for COVID-19 inpatient care significantly increased cost, hospital LOS, and inpatient admissions for displaced observation patients. As a result, this might have negative downstream effects, including inpatient bed availability, patient satisfaction, and the risk of nosocomial complications associated with prolonged hospital stays.

Observation patients are a well-defined hospital patient population who are managed in different hospital settings to determine their need for inpatient admission. Observation unit settings are defined by two variables: the use of a dedicated EDOU and observation protocols—with a type 1 setting using both, and a type 4 setting using neither. Observation patients in all hospitals with a type 1 unit will have "leakage” of observation patients to a type 4 setting. Our study focuses on the impact of a pandemic intervention on all hospital observation patients, not only the type 1/EDOU subset. We describe the impact of shifting observation patients from a type 1 setting to a type 4 setting to accommodate the pandemic. This population is most relevant to real-world hospitals faced with decisions regarding how to best leverage resources in a pandemic or disaster.

Among the 4 study hospitals, the intervention hospital had the highest proportion of observation patients managed in a type 1 setting during the pre-COVID period. Consequently, when all EDOU patients were shifted to a type 4 setting, the operational impact was most pronounced at this site. This made the hospital particularly well suited to evaluate the study objective. Transitioning observation patients away from a type 1 model may be associated with meaningful changes in hospital flow and resource utilization (Table S1).

Dedicated type 1 EDOUs offer protocol-based care aimed at reducing unnecessary admissions and streamlining patient flow. Prior studies have shown their efficacy in improving both clinical outcomes and financial performance.^1^^,^^12^, ^13^, ^14^ Other large-scale evaluations have demonstrated similar reductions in admission rates and health care costs through structured observation care.^14^

The repurposing of hospital resources, such as an EDOU, is not an uncommon strategy during public health emergencies. During the COVID-19 pandemic, many institutions adopted similar models, converting ambulatory or transitional care spaces into isolation units. Preparedness frameworks highlight the necessity of surge capacity planning to mitigate such consequences. Hasan et al’s^15^ systematic review emphasizes that maintaining modularity in hospital infrastructure, preserving spaces like EDOUs, is vital for sustainable emergency care delivery. The CHEST consensus statement further describes that the strategic allocation and preservation of intermediate acuity care areas are central to disaster readiness.^16^

The conversion of EDOUs was a well-intentioned measure to accommodate a surge of patients with COVID-19. However, as emphasized in disaster literature, such conversions should not eliminate intermediate care structures entirely. The CHEST consensus reports define surge capacity not simply as “more beds,” but as a structured approach to maintain layered care delivery.^16^

Our study findings provide a notable contrast with a study by Caspers,^17^ which also involved leveraging an EDOU in a disaster. However, in the Caspers study, there was an opposite approach to a disaster. They chose to open, rather than close, a type 1 EDOU, where one had previously not existed, with favorable results. In this study, 3 months after Hurricane Sandy had demolished the NYU Langone Medical Center hospital, an urgent care center was opened, and a single hospital floor was reopened as a type 1 EDOU. This allowed selected observation patients to avoid being admitted to regional hospitals, which were overwhelmed by this public health disaster. Over the following year, they managed 3,167 observation patients with common EDOU outcomes: 16.3 h LOS and 16% inpatient conversion rates, preserving scarce inpatient resources as New York City recovered. However, it should be acknowledged that these represent very different types of incidents. A natural disaster such as Hurricane Sandy primarily limited physical infrastructure, whereas the COVID-19 pandemic produced a surge of critically ill patients and markedly altered ED presentation patterns.

The implications suggest careful consideration when repurposing EDOUs during biological disasters, pandemics, or surge events. A review by Emeli et al^14^ shows that EDOUs can be rapidly adapted during chemical, biological, and environmental disasters to preserve ED flow, expand surge capacity, and protect inpatient beds, using several flexible operational models. This article describes 5 potential roles for leveraging an EDOU in a disaster, including displacing observation patients to use the EDOU for only disaster patients such as described in this study. The current study is the first to report the outcomes of that intervention. Across 7 real-world events, EDOUs consistently functioned as a critical “safety net” that enabled hospitals to maintain operations during crisis conditions.^18^ These findings highlight the need to balance immediate pandemic-response needs against the indirect impact on existing hospital populations, such as observation patients, and to develop alternative models of care, such as hospital-at-home and virtual observation units that could offer partial mitigation of these negative outcomes.

In terms of the generalizability of our findings, in any health system with a type 1 unit that is performing at benchmark, displacing EDOU patients to a type 4 setting to better serve disaster patient surges may come at a cost: increased LOS, cost, admit rate, and inpatient bed utilization for the displaced observation patients. If hospitals have a type 2 or type 3 observation unit, the effect demonstrated in this study may not be seen or may be represented differently. Future disaster preparedness should incorporate strategic planning for flexible EDOU utilization, but leaders should consider the impact on hospital observation outcomes. Displacing EDOU observation patients to inpatient beds to accommodate disaster or surge patients is associated with increases in observation patient cost, LOS, inpatient bed use, and inpatient admission rate.

## Author Contributions

MJC contributed to study design and wrote most of the manuscript. IE contributed to the introduction and discussion. MAR conceived the study and study design, contributed to the discussion, and edited for content, formatting, and grammar. TPM performed the statistical analysis, generated tables and figures, and contributed to the methods and results sections. DWC, GBH, MAWR, and IBB contributed to study design, and reviewed and edited the manuscript for content and grammar. All authors approved the final version of the manuscript.

## Funding and Support

Drs. Ross and Carr receive scholarship funding from the 10.13039/100019957National Foundation of Emergency Medicine (NFEM) to support their time and effort toward research productivity.

## Conflict of Interest

All authors have affirmed they have no conflicts of interest to declare.
