# Supplementary material for: The Impact of Leveraging an Emergency Department Observation Unit for Pandemic Response on Observation Outcomes: A Retrospective Observational Difference-In-Differences Study
Source: J Am Coll Emerg Physicians Open. 2026 Jul 28;7(5):100471. doi: 10.1016/j.acepjo.2026.100471 (PMC13430159; doi:10.1016/j.acepjo.2026.100471)
Supplement: Supplementary Tables 1 and 2 [file mmc1.docx]

**Supplemental Material**

**Supplement Table 1.** Percentage of Observation Patients Captured in ED or EDOU.

|  | Hospital  (#EDOU beds) | Pre-COVID | Post-COVID* |
| --- | --- | --- | --- |
| Intervention | EJCH  (11 beds) | 72.5  (70.9 - 74.2) | 40.2  (35.3 - 45.2) |
| Control | ESJH  (12 beds) | 42.6  (41.4 - 43.8) | 42.7  (39.9 - 45.6) |
|  | EUHM  (16 beds) | 52.3  (51.0 - 53.6) | 50.2  (47.2 - 53.3) |
|  | EUH  (13 beds) | 57.5  (56.0 - 59.0) | 56.5  (53.1 - 60.0) |
| Total control |  | 49.8  (49.1 - 50.6) | 49.0  (47.1 - 50.8) |

*In the Post-COVID period, psychiatric observation patients continued to be managed in the ED, accounting for 40.2% of all observation patients.

Abbreviations: Hospital names - blinded for reviewers.

**Supplemental Table 2.** Adjusted Study Outcomes for Only Community Hospitals

|  | **Pre** | **Post** | **Mean Ratio (95% CI)** | **p** |
| --- | --- | --- | --- | --- |
| **Sample Size** |  |  |  | - |
| ESJH | 6262 (894.6 / month) | 1170 (585 / month) | - | - |
| EJCH | 2687 (383.9 / month) | 393 (196.5 / month) | - | - |
| **Cost** |  |  |  | .03 |
| ESJH | 3405.4  (3346.3 – 3465.7) | 3714.9  (3566.8 – 3869.2) | 1.09  (1.04 – 1.14) | <.001 |
| EJCH | 3481.6  (3388.4 – 3577.4) | 4183.8  (3901.8 – 4486.3) | 1.20  (1.12 – 1.29) | <.001Tot |
| **Total LOS (obs + inpatient)** |  |  |  | .08 |
| ESJH | 36.3  (35.6 – 37.5) | 37.5  (35.8 – 39.2) | 1.03  (0.98 – 1.08) | .23 |
| EJCH | 32.6  (31.7 – 33.6) | 36.7  (33.9 – 39.6) | 1.12  (1.03 – 1.22) | .01 |
| **Obs LOS** |  |  |  | <.001 |
| ESJH | 22.2  (21.8 – 22.6) | 20.8  (19.9 – 21.7) | 0.94  (0.89 – 0.98) | .01 |
| EJCH | 21  (20.4 – 21.6) | 25.2  (23.4 – 27.1) | 1.20  (1.11 – 1.30) | <.001 |

Abbreviations: Obs (Observation); LOS (Length of Stay); Hospital names - blinded for reviewers.
